# Transposon mutagenesis screen in mice identifies *TM9SF2* as a novel colorectal cancer oncogene

**DOI:** 10.1038/s41598-018-33527-3

**Published:** 2018-10-17

**Authors:** Christopher R. Clark, Makayla Maile, Patrick Blaney, Stefano R. Hellweg, Anna Strauss, Wilaiwan Durose, Sambhawa Priya, Juri Habicht, Michael B. Burns, Ran Blekhman, Juan E. Abrahante, Timothy K. Starr

**Affiliations:** 1 Department of Obstetrics, Gynecology and Women’s Health, University of Minnesota, Minneapolis, MN USA; 2 Masonic Cancer Center, University of Minnesota, Minneapolis, MN USA; 3 University of Minnesota Informatics Institute, Minneapolis, MN USA; 4 Department of Genetics, Cell Biology, and Development, University of Minnesota, Minneapolis, MN USA; 5 Department of Ecology, Evolution, and Behavior, University of Minnesota, Minneapolis, MN USA; 6 Department of Biology, Loyola University, Chicago, IL USA

## Abstract

New therapeutic targets for advanced colorectal cancer (CRC) are critically needed. Our laboratory recently performed an insertional mutagenesis screen in mice to identify novel CRC driver genes and, thus, potential drug targets. Here, we define *Transmembrane 9 Superfamily 2* (*TM9SF2*) as a novel CRC oncogene. TM9SF2 is an understudied protein, belonging to a well conserved protein family characterized by their nine putative transmembrane domains. Based on our transposon screen we found that *TM9SF2* is a candidate progression driver in digestive tract tumors. Analysis of The Cancer Genome Atlas (TCGA) data revealed that approximately 35% of CRC patients have elevated levels of *TM9SF2* mRNA, data we validated using an independent set of CRC samples. RNAi silencing of TM9SF2 reduced CRC cell growth in an anchorage-independent manner, a hallmark of cancer. Furthermore, CRISPR/Cas9 knockout of *TM9SF2* substantially diminished CRC tumor fitness *in vitro* and *in vivo*. Transcriptome analysis of *TM9SF2* knockout cells revealed a potential role for TM9SF2 in cell cycle progression, oxidative phosphorylation, and ceramide signaling. Lastly, we report that increased *TM9SF2* expression correlates with disease stage and low *TM9SF2* expression correlate with a more favorable relapse-free survival. Taken together, this study provides evidence that *TM9SF2* is a novel CRC oncogene.

## Introduction

Colorectal cancer (CRC) arises from a stepwise accumulation of mutations that transform normal epithelia into cancerous tissue^1,2^. Decades of research analyzing the genetic basis of CRC has resulted in the identification of several important driver genes including *APC, KRAS, SMAD4*, and *TP53*. In addition, recent large scale genomic analyses, such as The Cancer Genome Atlas (TCGA), have identified numerous additional recurrent somatic mutations, focal copy number alterations and gene expression changes^3,4^. From these studies it is clear that CRC has a complex genetic etiology. It is important that we understand the functional significance of these genetic changes so that we can develop better therapies, especially for advanced disease.

To functionally define genetic drivers of CRC, we and others have used *Sleeping Beauty* (SB) transposon mutagenesis screens in mice, an unbiased method of finding genetic drivers of CRC. These studies have produced multiple lists of genes suspected of contributing to CRC when altered by transposon mutagenesis^5–8^. With the goal of finding potential therapeutic targets we are using cross-species bioinformatics approaches to select genes from these lists for further study. This approach has resulted in the identification of potential actionable targets including *KCNQ1*, *CFTR*, and *RSPO2/3*^9–11^. In this study, we report our findings on *TM9SF2*, a transmembrane protein belonging to the transmembrane-9 superfamily (TM9SF), which includes *TM9SF1-4*. Although it is well conserved evolutionarily, very little is known about the function of TM9SF proteins in mammalian cells, nor their role in cancer^12,13^. *TM9SF1* has been implicated in autophagosome formation and has been linked to bladder cancer^14,15^. It has been reported that *TM9SF3* is upregulated in chemoresistant breast cancer cells after combination treatment with paclitaxel and an HDAC inhibitor and may also play a role in gastric cancer^16,17^. The most well studied member, TM9SF4, is reportedly overexpressed in human melanoma cells and has also been described as a proton pump associated protein^18,19^.

In this study, we identify *TM9SF2* as a novel oncogene in CRC. We found that *TM9SF2* is potentially regulated by the Ets-family transcription factor *ELF1*, and *TM9SF2* is upregulated in approximately one-third of human CRC samples. We used RNAi and CRISPR/Cas9 to either reduce or knockout the expression of *TM9SF2*, which had the effect of reducing tumor fitness in both the *in vitro* and *in vivo* settings. Finally, we performed transcriptome analysis to gain insight into the potential role of *TM9SF2* as a cell cycle regulating protein.

## Results

### Insertional mutagenesis screens identify *TM9SF2* as candidate cancer gene

**Figure 1:**
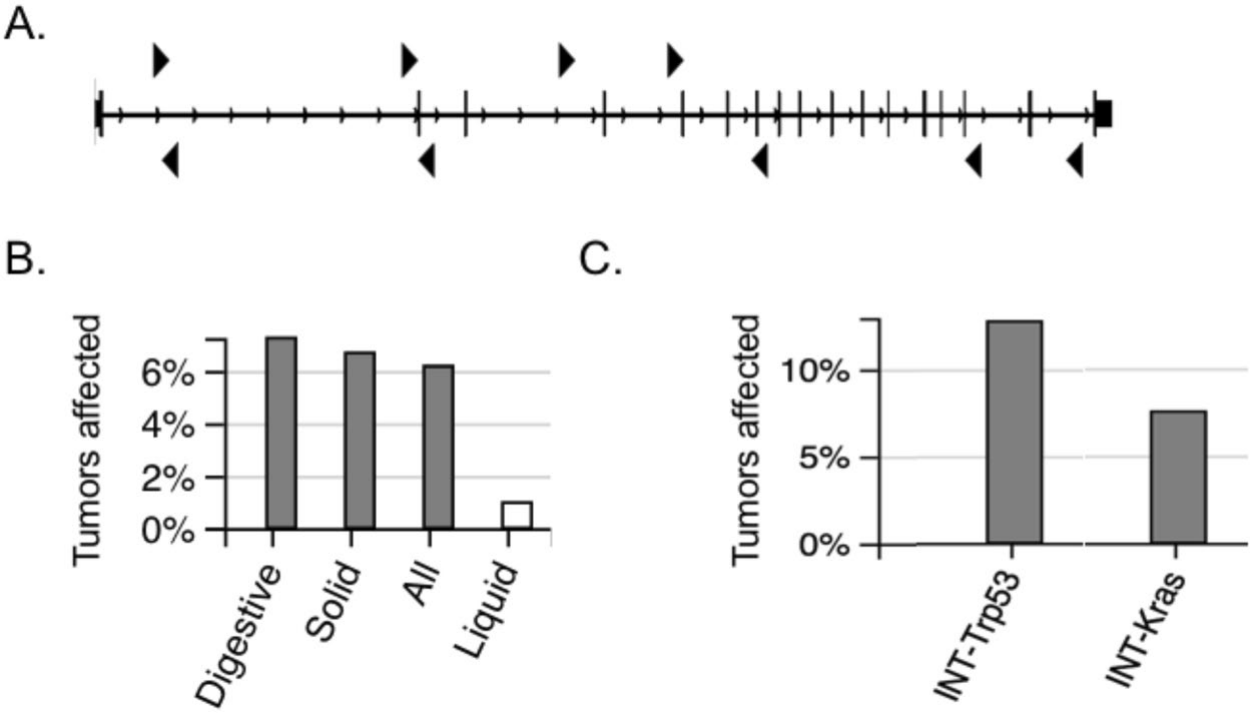
SB screen identifies TM9SF2 as candidate CRC driver gene. *Tm9sf2* is a CIS gene in SB transposon screens. (**A**) schematic representation of gastrointestinal tract tumor-T2/onc insertion sites within the murine *Tm9sf2* gene. Triangles depict the location of insertion as well as the orientation of the promoter-splice donor within the transposon. (**B**) The frequency of tumors with SB insertions in *Tm9sf2* in digestive tract, solid tumor, liquid tumors, and all tumors analyzed in the SBCD database. Gray bars represented instances where *Tm9sf2* is a progression diver gene. White bars are not significantly altered cases. (**C**) The frequency of *Tm9sf2* insertions in intestinal-specific mutagenesis screens in mice with predisposing mutations in *Trp53* (R172H allele) or *Kras* (G12D allele). *Tm9sf2* insertions are predicted to act as a progression driver gene in both studies.

Our laboratory previously performed an insertional mutagenesis screen in mice to identify novel gastrointestinal (GI) tract cancer driver genes^5^. In this study we used the *Sleeping Beauty* (SB) DNA system consisting of an oncogenic DNA transposon (T2/Onc) capable of disrupting tumor suppressor genes and activating oncogenes, which is activated by tissue-specific expression of the SB transposase^20–22^. We identified 77 candidate cancer genes whose activity was potentially altered by *T2/Onc* transposition based on common insertion site (CIS) analysis^23^. Of these 77 candidate cancer genes, we chose to focus on *TM9SF2* for further study because we found this gene to be overexpressed in a large percentage of human CRC samples, suggesting a potential oncogenic function. *TM9SF2* is a member of a highly conserved family of proteins that span the lipid bilayer nine times. The predicted function of the *TM9SF2* protein product is to act as a small molecule transporter or ion channel. In our screen the *T2/Onc* transposon insertions were mapped to the murine *Tm9sf2* gene in nine tumor samples (Fig. 1A).

To further explore the role of TM9SF2 as a cancer gene, we used two publicly available databases that catalog cancer genes discovered using DNA transposon insertional mutagenesis. The Candidate Cancer Gene Database (CCGD, http://ccgd-starrlab.oit.umn.edu/about.php) catalogs cancer genes identified in 69 insertional mutagenesis studies covering 12 tumor types^8^. Mining the CCGD database revealed that *Tm9sf2* was a transposon-targeted mutation in an additional eight forward genetic screens, including screens for liver, pancreatic, breast, and gastric cancer (see Supplementary Table S2). The Sleeping Beauty Cancer Driver Database (SBCDDB: http://sbcddb.moffitt.org/index.html) catalogs over 1.5 million transposon insertions from 2 354 tumors taken from approximately 1 000 mice from 19 tumor types^24^. Mining of the SBCDDB revealed that *Tm9sf2* was a common insertion site in 7.2% (121/1674 tumors) of all digestive tumors, which includes liver, pancreas, intestine, and stomach tumors, but was not identified as a driver in hematopoietic tumors (Fig. 1B). Several insertional mutagenesis studies were conducted using mice that are predisposed to GI tract cancer by manipulating known genetic drivers, including *Apc*, *Kras*, *Smad4*, and *Trp53*^6,7,25^. Interestingly, *TM9SF2* was not identified as a driver gene in mice with *Apc* or *Smad4* mutations but was identified as a driver in mice with *Kras* or *Trp53* mutations. Mice harboring the activating *Kras* G12D allele had *Tm9Sf2* transposon insertions in 13 out of 173 tumors (7.5%; p = 1.68e-05) and mice harboring the dominant negative R172H *Trp53* mutation had insertions in 7 out of 55 tumors (12.7%; p < 0.005) (Fig. 1C and Supplementary Table S3). This analysis indicates TM9SF2 has a contributing role in the formation of murine intestinal tumors.

### *TM9SF2* is overexpressed in human CRC

To determine if *TM9SF2* is altered in human disease we evaluated *TM9SF2* mutation data from the Catalogue of Somatic Mutations in Cancer (COSMIC) database (see: http://cancer.sanger.ac.uk/cosmic/publications)^26^. Interestingly, data from COSMIC revealed that mutations in *TM9SF2* are rarely catalogued in human tumors. We found that tumors derived from the endometrium contained the highest rate of *TM9SF2* mutation at a tumor frequency of approximately 2% (13/656 total tested). Tumors from the small and large intestine were the next most likely to contain mutations in *TM9SF2* but the mutation frequency remained very low at 1.92% and 1.71% respectively. These data suggest that point mutations and small in/dels in *TM9SF2* are unlikely to play a significant role in CRC development and progression.

**Figure 2:**
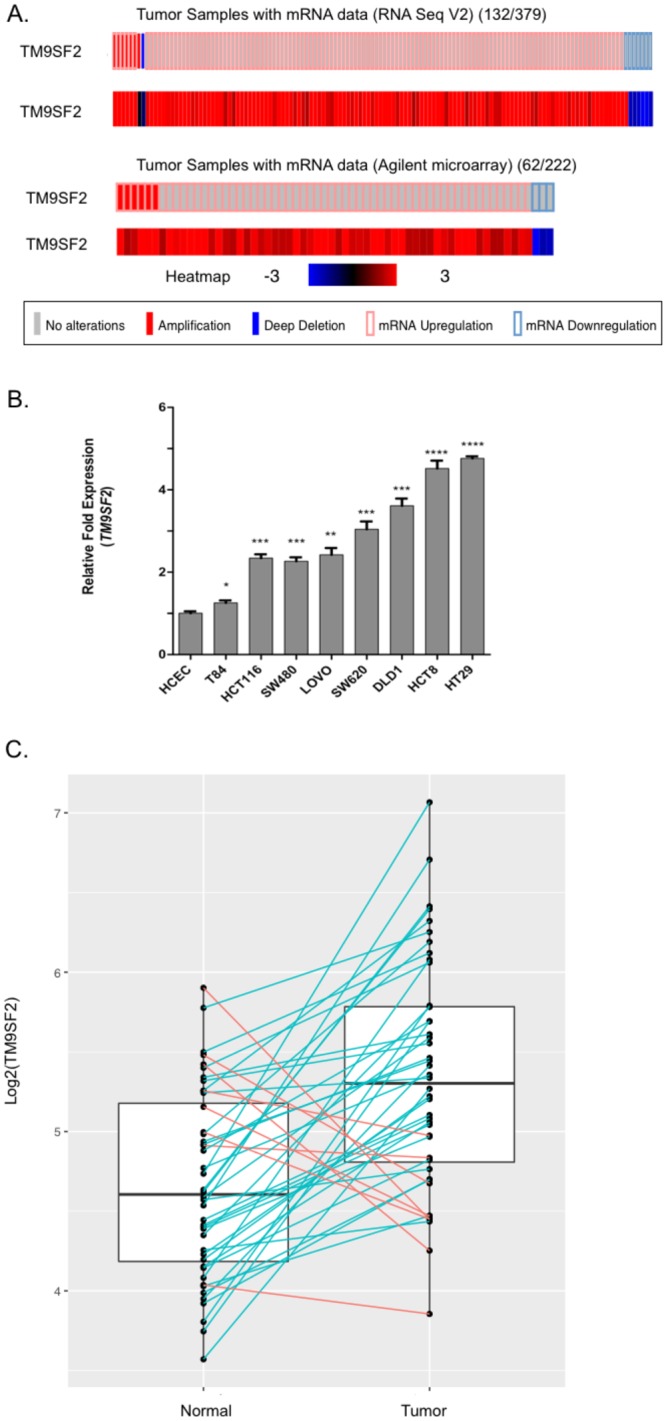
TM9SF2 is overexpressed in CRC cell lines and patient samples. qRT-PCR and transcriptomics demonstrate *TM9SF2* overexpression in CRC cell lines and patient derived samples. (**A**) (top) Oncoprint of genomic alterations in 379 TCGA patient colorectal adenocarcinomas subjected to RNA sequencing. *TM9SF2* copy number alterations and mRNA expression changes are shown in addition to a heatmap depicting the intensity of *TM9SF2* mRNA changes. (**A**) (bottom) Oncoprint showing alterations in *TM9SF2* in 222 TCGA patients subjected to Agilent microarray analysis. (**B**) qRT-PCR analysis for *TM9SF2* expression in human CRC cell lines. HCEC, an immortalized but non-transformed colon epithelial cell line, was used as the control. (**C**) RNA sequencing results showing *TM9SF2* expression levels in a set of CRC tumor and matched normal tissue samples from the University of Minnesota. Green lines highlight samples with an increase in *TM9SF2* mRNA in tumor versus normal and red lines indicate a decrease. Expression differences between healthy and tumor tissue were tested for significance using non-parametric Mann-Whitney U test (paired). T-test *P < 0.05. **P < 0.01. ***P < 0.001. ****P < 0.0001.

We used several approaches to measure *TM9SF2* gene expression in CRC. First, we used cBioportal to analyze gene expression in CRC tumors from The Cancer Genome Atlas^27,28^. Analyses of gene expression levels using microarray or RNA sequencing reveal that, when compared to the mean expression distribution of tumor samples that are diploid for *TM9SF2*, *TM9SF2* is overexpressed in approximately one-third (194/601 tumors) of large intestine tumors (Fig. 2A). These data suggest that *TM9SF2* may function as a proto-oncogene in CRC. For our second approach, we evaluated the mRNA levels of *TM9SF2* in a panel of nine commonly used colorectal cancer cell lines using qRT-PCR. For comparison we used the Human Colonic Epithelial Cell (HCEC) line, which is a non-oncogenic immortalized cell line, as a normal (non-tumor) control sample^29^. All cell lines tested had significantly upregulated levels of *TM9SF2* transcript when compared to HCEC. DLD1, HCT-8, and HT-29 cells expressed the highest levels of *TM9SF2* ranging from approximately 4 to 5-fold that of HCEC cells (Fig. 2B). Finally, we performed RNA-sequencing on a set of 44 CRC tumor and matched normal samples collected at the University of Minnesota. Expression levels were significantly increased in tumors compared to matched normal samples (P = 4.461 × 10^−6^; Fig. 2C). In all but eight samples the levels of *TM9SF2* mRNA were increased in the patient’s tumor sample compared to the matched normal sample (Fig. 2C). These data support the hypothesis that *TM9SF2* is overexpressed in CRC and may play a role as an oncogene.

### *TM9SF2* functions as an oncogene in CRC cell lines

**Figure 3:**
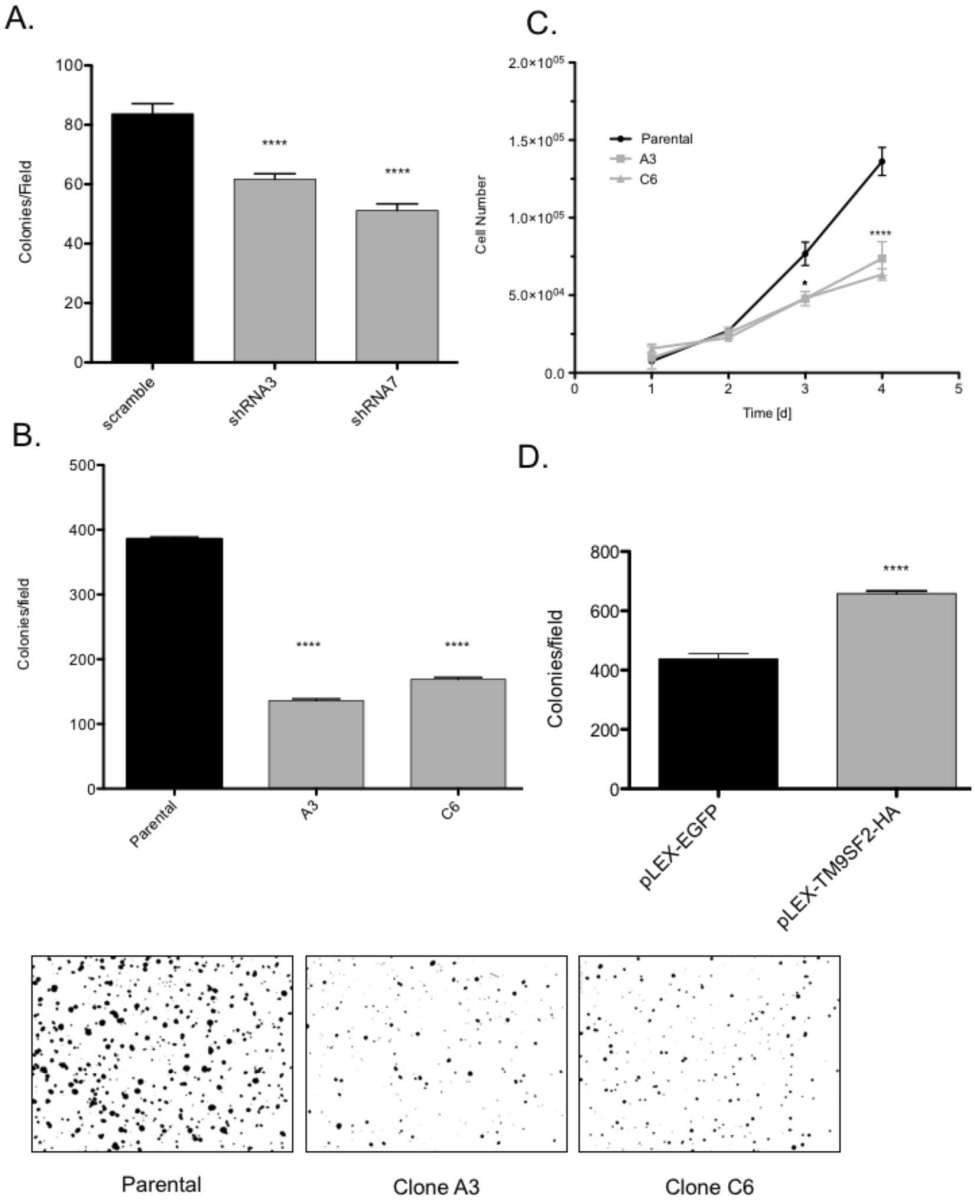
CRISPR/Cas9 knockout of TM9SF2 reduces cellular proliferation rate and colony formation *in vitro*. CRISPR/Cas9 mediated deletion of *TM9SF2* reduces cellular proliferation and anchorage-independent cell growth. (**A**) Quantification of colonies indicating that *TM9SF2* knockdown reduces DLD1 growth in soft agar. Two independent knockdown clones, sh3 and sh7, were used in this experiment. (**B**) Representative quantification of colonies indicating that *TM9SF2* knockout reduces cell growth in soft agar. Two independent single cell clones, A3 and C6, were used in this experiment. (**B**) Images of colonies stained with crystal violet ten days post plating. (**C**) proliferation assay measuring trypan blue exclusion in parental (black line) and TM9SF2 HT-29 knockout clones (gray lines). (**D**) Representative quantification of colonies indicating TM9SF2 overexpression increases cell growth in soft agar. T-test *P < 0.05. T-test ****P < 0.0001.

To determine if *TM9SF2* plays an oncogenic role in CRC, we generated stable *TM9SF2* knockdowns in DLD1 cells using lentiviral shRNA vectors (Fig. S1A,B). Reducing the level of TM9SF2 in DLD1 cells resulted in reduced anchorage-independent growth compared to empty-vector control cells based on their reduced ability to form colonies in soft agar. We observed a significant reduction in colony numbers in two independent knockdown DLD1 cell lines (shRNA3: 25% and shRNA7: 37% (Figs 3A and S1C).

To further validate the oncogenic role of TM9SF2, we used CRISPR/Cas9 editing to knockout the *TM9SF2* gene in HT-29 and HCT116 CRC cell lines (Supplementary Fig. 2). We generated multiple independent clones that did not express TM9SF2 based on sequencing and Western blot analysis (Supplementary Fig. S2D). Similar to the DLD1 knockdown cell line, loss of TM9SF2 in HT-29 resulted in reduced anchorage-independent growth based on the soft agar assay (Fig. 3B). In addition, the proliferation rate was decreased in HT-29 TM9SF2 KO cells (Fig. 3C). Anchorage-independent growth and proliferation were not affected in *TM9SF2* knockout HCT116 cells (data not shown), which is likely due to the already low level of expression in the parental HCT116 cells (Fig. 2B). In fact, overexpression of *TM9SF2* in HCT116 cells resulted in increased colony growth in soft agar, which further supports the finding that *TM9SF2* functions as a CRC driving oncogene (Figs 3D and S1E).

### *TM9SF2* functions as an oncogene *in vivo*

**Figure 4:**
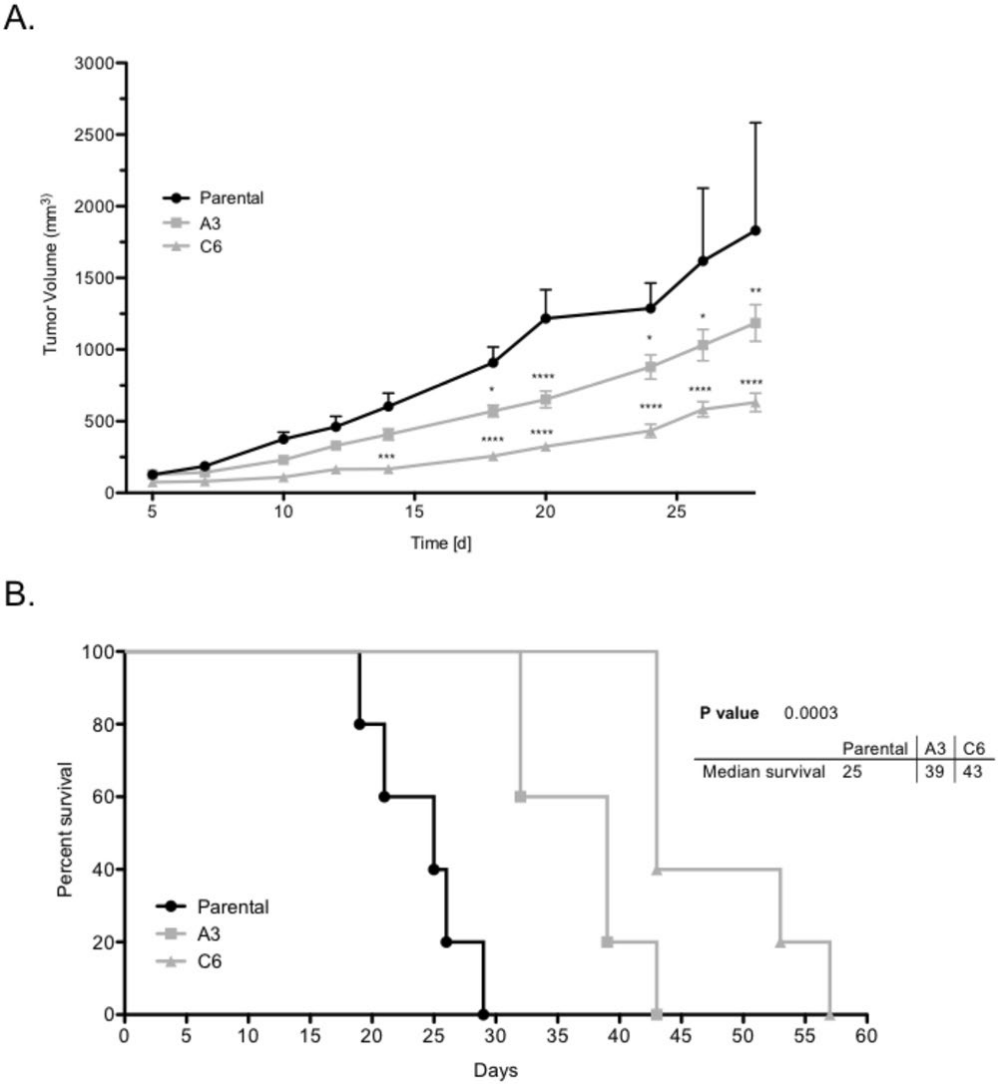
TM9SF2 knockout reduces tumor growth and prolongs survival in a xenograft model. *TM9SF2* knockout reduces tumor growth and extends survival in an *in vivo* xenograft model. (**A**) HT-29 TM9SF2 knockout cells were injected subcutaneously in the rear flank of athymic nude mice and tumor volume was measured with calipers approximately every other day. *TM9SF2* knockout cells have a significantly reduced ability to grow versus control cells. (**B**) Kaplan-Meier survival curves for control animals and two groups bearing tumors from two independent TM9SF2 knockout clones. *P < 0.05. **P < 0.01. T-test ****P < 0.0001.

To determine if *TM9SF2* knockout has an effect on tumor growth *in vivo* we performed a xenograft experiment using HT-29 *TM9SF2* KO cells compared to parental cells. By day 20, mean tumor volume for parental control cells exceeded 1 000 mm^3^ compared to 600 mm^3^ and 325 mm^3^ for knockout clones A3 and C6 respectively (Fig. 4A). This reduced tumor burden corresponded to a significantly increased overall survival for mice bearing *TM9SF2* knockout tumor cells. Mice grafted with clones A3 and C6 had a median survival of 39 and 43 days, while mice grafted with control cells survived on average only 25 days (Fig. 4B). Taken together, these data support the hypothesis that *TM9SF2* functions as an oncogene in CRC.

### Cell cycle and metabolic pathways upregulated by *TM9SF2*

To uncover the molecular pathways affected by TM9SF2 we performed RNASeq and quantified transcript levels in the parental and TM9SF2 knockout HT-29 cells. We identified 835 genes that were differentially expressed, with 596 being increased and 239 being decreased in the knockout cells (see Supplementary Table S5). The expression of several notable cell cycle checkpoint genes including *CCNA2*, *CCNB2*, and *AURKA*, as well as the Myc target proliferation-related genes *PCNA* and *NPM1*, were all significantly lower in the *TM9SF2* knockout cells. The reduced proliferative phenotype observed in *TM9SF2* knockout cells can likely be explained by the downregulation of these genes.

**Figure 5:**
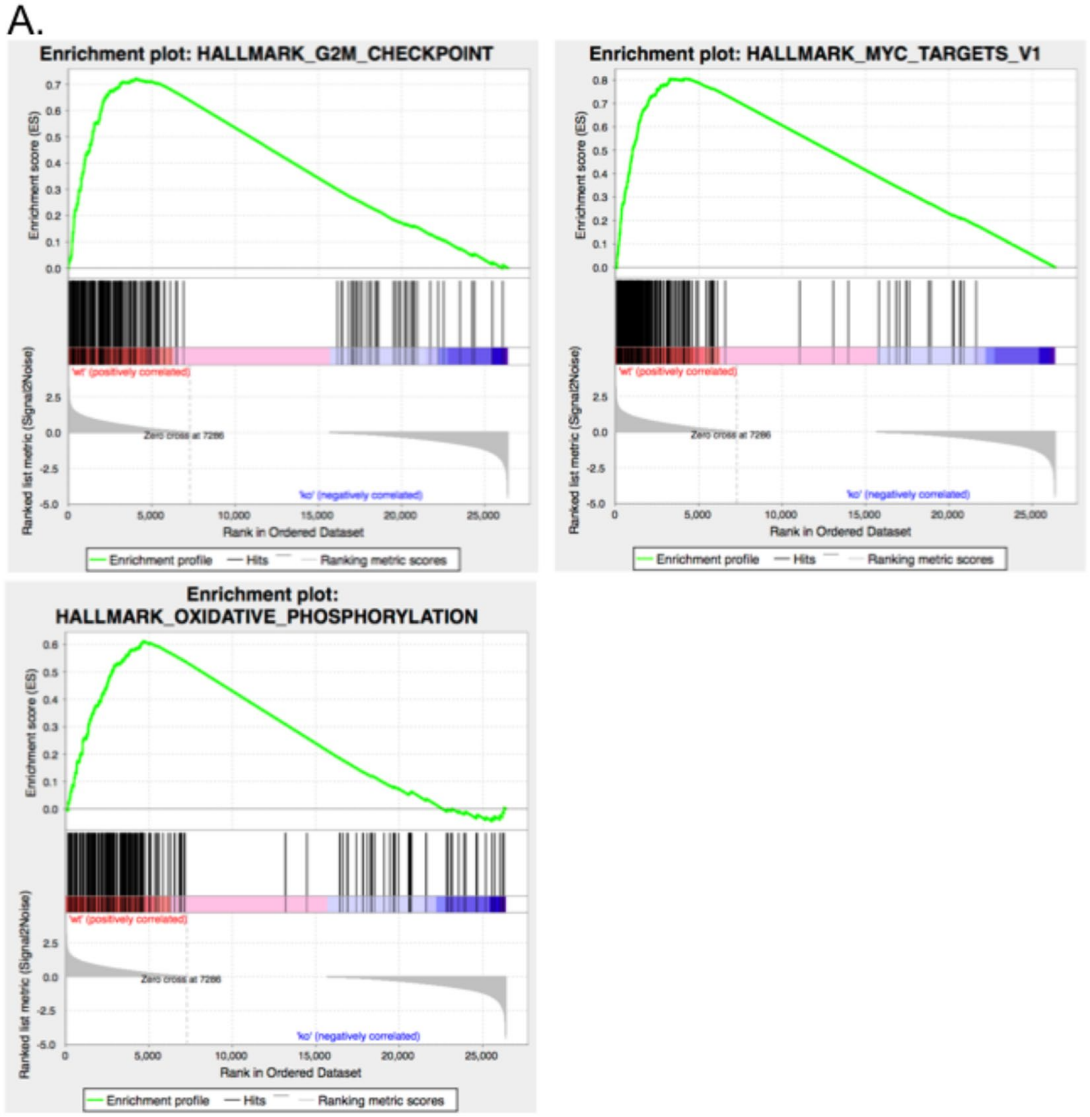
TM9SF2 is a potential cell cycle regulator. *TM9SF2* knockout demonstrates its role as a potential cell cycle regulator. Three panels showing the enrichment score (ES) for the top three hallmark gene sets enriched in the control HT-29 cells compared to the TM9SF2 knockout cells.

To identify gene sets that are affected by *TM9SF2* loss we performed Gene Set Enrichment analysis (GSEA) on the set of altered genes^30^. Gene sets that were significantly enriched in parental controls included the G2-M cell cycle checkpoint genes, Myc target genes, and genes in the reactive oxygen species pathway (Fig. 5 and Table 1). The highest-ranking member of the reactive oxygen species pathways was the glucose-6-phosphate dehydrogenase gene (*G6PD*). Loss or downregulation of this gene has been known to cause embryonic lethality in mice and lead to cellular senescence in various cell types^31–33^. *TM9SF2* knockout cells have more than a two-fold decrease in *G6PD*, which likely contributes to the reduced oncogenic behavior of these cells.

**Table 1:** Genesets enriched in parental HT-29 cells compared to HT-29 TM9SF2 knockout cells.

Geneset	NES	p-value	FDR q-val
Hallmark_G2M CHECKPOINT	1.25	0.000	0.215
Hallmark_MYC TARGETS V1	1.26	0.000	0.246
Hallmark_Oxidative Phosphorylation	1.3	0.0063	0.252

We used Ingenuity Pathway Analysis to identify canonical pathways altered in *TM9SF2* knockout cells. This analysis found significant alterations in the expression of genes associated with Ceramide signaling, Mitotic Roles of Polo-like Kinase, and Protein Kinase A signaling pathways (Supplementary Table S6). Notable Ceramide signaling genes altered in the HT-29 knockout cells included *PIK3R3* (−2.1-fold)*, S1PR2* (+5.7-fold)*, SMPD3* (−8.9-fold)*, and SPHK1* (+5.2-fold). These genes, and the majority of the other molecules associated with Ceramide signaling, are altered in the direction consistent with pathway activation. These genes are known to play an integral part in the metabolism of Sphingolipids and they have been associated with various neoplasms^34–38^.

### *ELF1* transcription factor regulates *TM9SF2* expression

**Figure 6:**
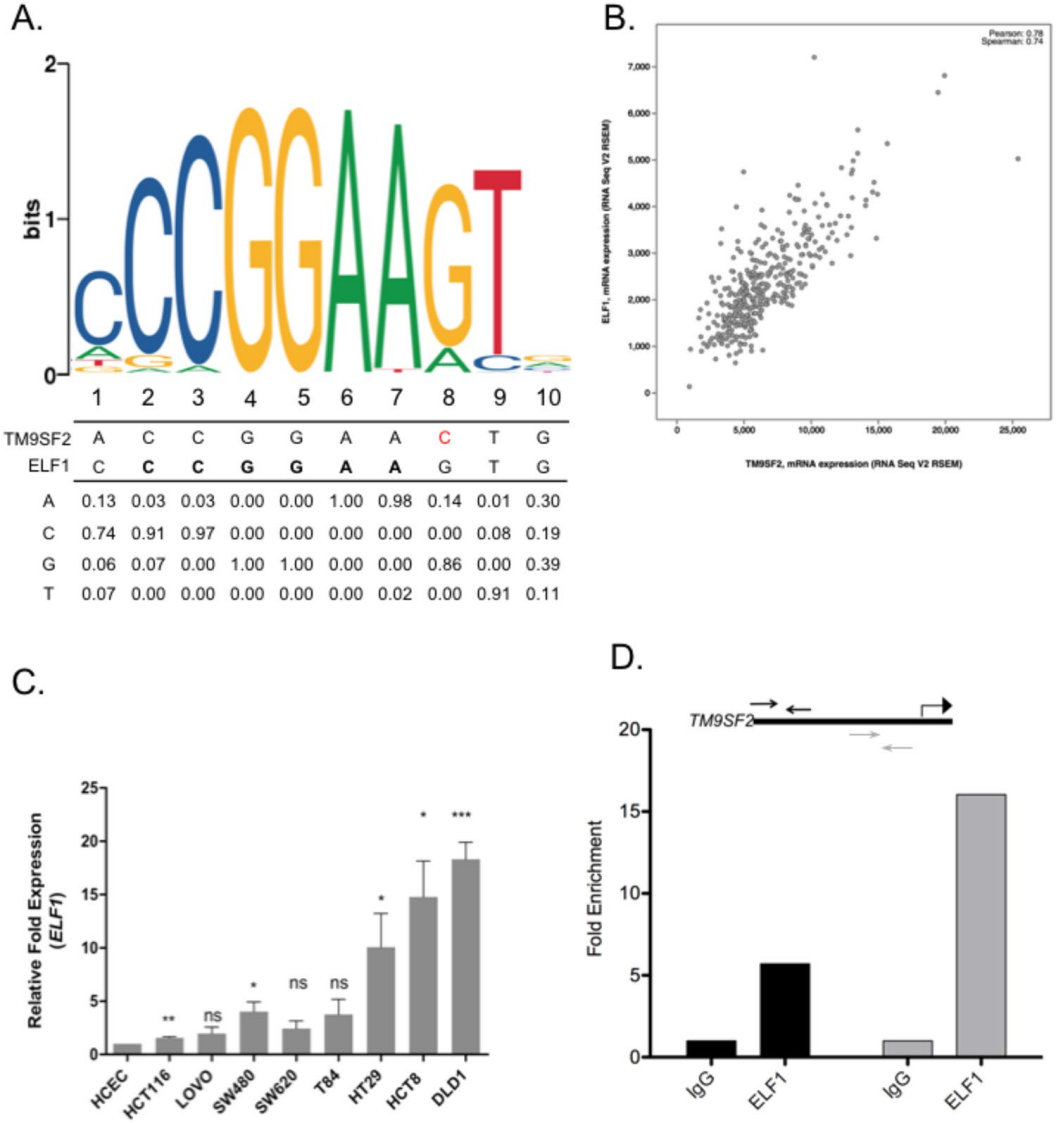
The Ets family transcription factor ELF1 regulates TM9SF2 expression. *ELF1* is a potential regulator of *TM9SF2* gene expression. (**A**) Position weight matrix from ENCODE CHIP-seq experiments showing a potential ELF1 transcription factor binding motif in the 5′ UTR of the TM9SF2 gene. (**B**) *ELF1* and *TM9SF2* mRNA co-expression scatter plot using TCGA RNAseq data from CRC patients. (**C**) qRT-PCR analysis for *ELF1* expression in human CRC cell lines. HCEC, an immortalized but non-transformed colon epithelial cell line, was used as the control. (**D**) Data from *ELF1* ChIP normalized against those from IgG mock ChIP. Schematic shows binding location of two qPCR primer sets (black and gray arrows) used to quantify TM9SF2 promoter DNA after ELF1 ChIP. Translation start site is indicated with bent arrow. t-test, ns = not significant, *P < 0.05. **P < 0.01. ***P < 0.001. ****P < 0.0001.

To identify regulatory factors responsible for upregulation of *TM9SF2* in CRC we analyzed HCT116 CHIP-Seq data from the ENCODE project^39^. Based on ENCODE data, there was a strong ELF1 binding motif (chr13: 100,153,732–100,153,742) in the 5′UTR of *TM9SF2*. The consensus ELF1 binding motif is 5′-CGGAAGT, which is a near perfect sequence match to the observed ELF1 DNA binding site in the *TM9SF2* promoter region (5′-CGGAACT). Based on ENCODE Chip-seq data, the location of the ELF1 binding site overlaps with elevated levels of H3K4 trimethylation, an epigenetic mark commonly associated with promoters (Figs 6A and S3).

If *ELF1* is required for elevated expression of *TM9SF2*, we predicted that *ELF1* mRNA levels would correlate with *TM9SF2* expression. To test this prediction, we analyzed *ELF1* and *TM9SF2* mRNA expression levels in 382 TCGA CRC patient samples and found a strong positive correlation (Pearson: 0.781; Spearman 0.744) between the expression of *TM9SF2* and *ELF1* (Fig. 6B). For further validation we performed qRT-PCR for *ELF1* in our panel of CRC cell lines. *ELF1* expression remains generally unchanged in the five cell lines with lower *TM9SF2* expression but is highly upregulated in the three cell lines with the highest *TM9SF2* expression (HT-29, HCT-8 and DLD1) (Figs 2B and 6C).

To verify that ELF1 binds to the *TM9SF2* promoter we performed CHIP-qPCR using DLD1 cells. At steady state we observed a 6-15-fold enrichment in *TM9SF2* promoter DNA after chromatin pull-down with an ELF1 antibody (Fig. 6D). These data are in support of *ELF1* as a *TM9SF2* regulating transcription factor.

### High levels of *TM9SF2* correlate with higher stage cancer and decreased disease-free survival

**Figure 7:**
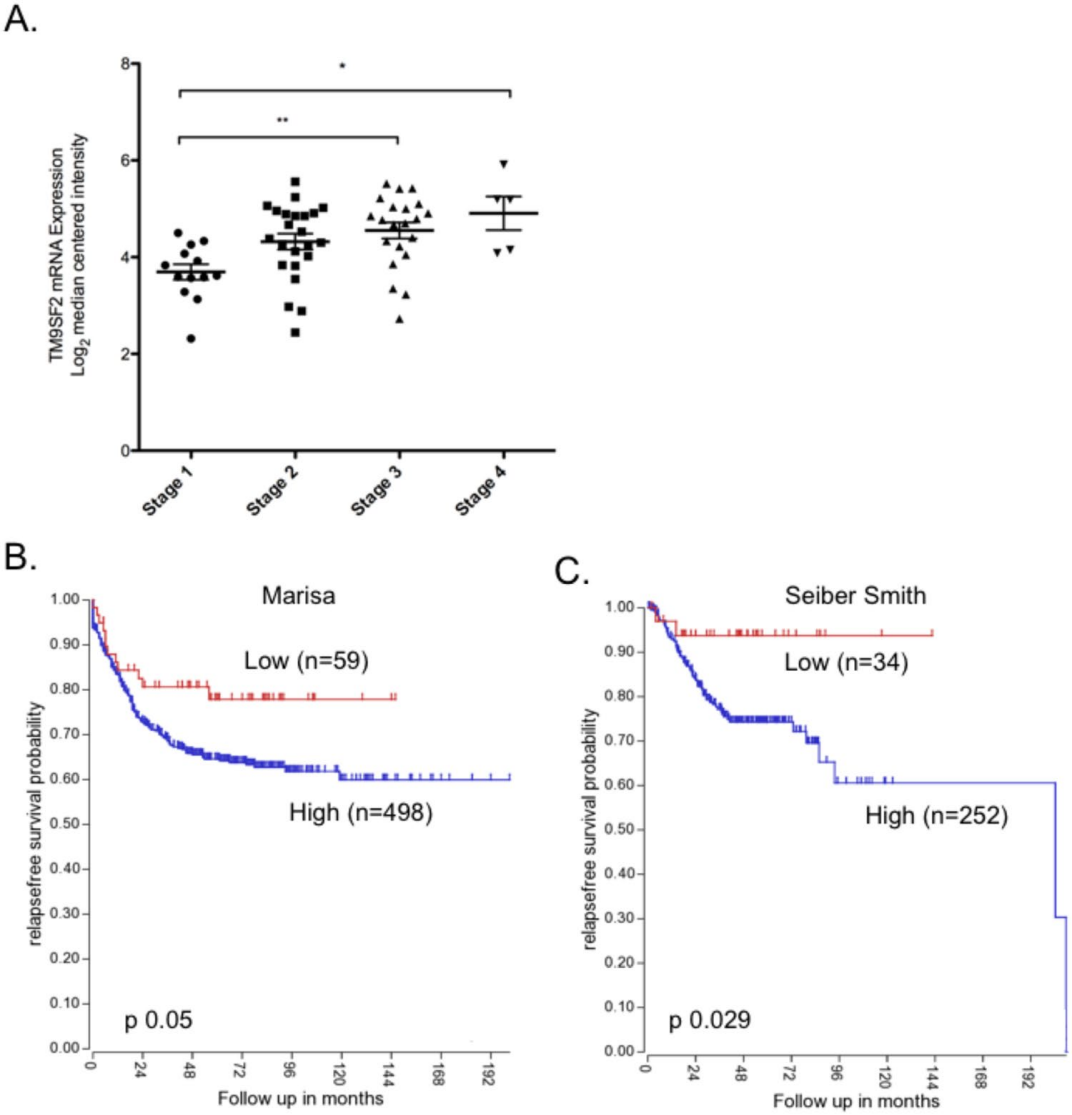
TM9S2 expression correlates with stage and predicts relapse free survival. *TM9SF2* expression correlates with disease stage and predicts relapse-free survival. (**A**) Microarray data (Staub, 2009, GSE12945) depicting *TM9SF2* mRNA expression level in patient samples from stages I through IV. (**B**,**C**) Kaplan-Meier curves depicting relapse-free survival with data stratified by intensity of *TM9SF2* mRNA measured in CRC samples via microarray (Left, Marissa, 2013, GSE39582, Right, Sieber Smith, 2010, GSE14333 plus GSE17538 minus identical samples).

Based on our data supporting a role for *TM9SF2* as an oncogene in CRC we tested the hypothesis that *TM9SF2* expression correlates with worse patient prognosis. We analyzed data from Staub *et al*., (Staub, 2009, GSE12945) where they measured gene expression in 62 patient samples spanning all clinical stages of CRC (I-IV). We found that *TM9SF2* expression correlates with disease severity with late stage (III & IV) cancers having a significantly higher level of expression compared to stage I (Fig. 7A).

In two additional studies with 566 patients (Marisa *et al*.) and 355 patients (Sieber *et al*.; Smith *et al*.) we tested the association of TM9SF2 mRNA levels with disease-free survival^40–42^. In both studies, we observed a significantly favorable disease-free survival probability in patients with the lowest levels of *TM9SF2* expression (Fig. 7B,C).

## Discussion

Our studies support the hypothesis that *TM9SF2* functions as an oncogene in CRC. Analysis of data from our previous SB transposon forward genetic screen identified *Tm9sf2* as a top candidate cancer gene. Mining of other publicly available SB transposon databases revealed further support for *Tm9sf2* as a candidate cancer gene. Transposon insertions in the murine *Tm9sf2* gene occurred in over 7% of the 1 674 analyzed digestive tract tumors and is predicted to be a progression driver gene^24^. We demonstrated using both TCGA data and our own independent data set that *TM9SF2* mRNA is overexpressed in up to one-third of CRC patients. Finally, using RNAi and CRISPR/Cas9 gene editing we demonstrated that reduction or complete knockout of TM9SF2 resulted in reduced tumor fitness in the *in vitro* and *in vivo* settings.

Our data also support *TM9SF2* expression as a potential prognostic indicator as we found that mRNA expression levels correlate with both disease stage and relapse-free survival probability. We observed a significant increase in *TM9SF2* expression in patients with stage III/IV disease versus those with stage I, suggesting that *TM9SF2* expression may promote either the migration of cancerous cells or their seeding and growth in distant organs.

The molecular mechanism of TM9SF2’s oncogenic function in CRC carcinogenesis is currently unknown. Others have demonstrated that TM9SF proteins are responsible for controlling surface expression of adhesion molecules^43,44^. In our RNA-seq data we observed alterations in several cell adhesion related genes including *ITGA1* and three members of the *CEACAM* family (*CEACAM5, CEACAM6, CEACAM19*). The mRNA levels of *CEACAM5* and *CEACAM6* were reduced by 4.9-fold and 12.3-fold respectively in knockout cells. Both *CEACAM5* and *CEACAM6* are known to play a critical role in facilitating tumorigeneis and metastasis by inhibiting anoikis^45,46^. The reduced ability of *TM9SF2* knockout cells to grow in soft agar could be explained by an increased sensitivity to anoikis due to the accompanying decrease in CEACAM expression.

It was recently reported that TM9SF4 plays a role in the assembly of the V-ATPase proton pump in CRC cells^19^. Silencing of *TM9SF4* expression led to a more acidic cytoplasmic pH with an accompanying alkalization of intracellular vesicles. Ingenuity pathway analysis revealed that the top canonical pathway altered in *TM9SF2* knockout cells was the Ceramide Signaling pathway. Ceramides are bioactive lipids that are critical for modulating multiple cellular processes including cell cycle, apoptosis, senescence, and stemness^47^. Ceramide levels within cells are tightly controlled by pH sensitive phospholipases called sphingomyelinases^48^. With a close similarity in amino acid sequence and structure to TM9SF4, we hypothesize that TM9SF2 may function as a membrane-bound vesicular protein involved in the regulation of intracellular acidity. Future studies are required to determine the role of TM9SF2 in intracellular ion homeostasis and regulation of ceramide signaling molecules.

An increased understanding of the genetic basis of CRC will be useful for designing new targeted therapies. Current efforts to target the known drivers of CRC, including *APC*, *SMAD4*, *TP53*, and *KRAS*, have not yet resulted in significant improvements in survival for advanced CRC. In the current study we identify *TM9SF2* as an oncogene that is most likely regulated by the transcription factor *ELF1*. Our findings provide a rationale for exploring the efficacy of drugs targeting either TM9SF2 or ELF1 for treating advanced-stage CRC.

## Materials and Methods

All experiments were performed in accordance with relevant guidelines and regulations.

### Cells

All cell lines (DLD1, LoVo, HCT116, HT-29, HCT-8, SW480, SW620, and T84) except HCEC were obtained from the ATCC and were minimally passaged. Human colonic epithelial cells (HCEC) were immortalized by expression of cyclin dependent kinase 4 (Cdk4) and the active components of human telomerase (hTERT) and were kindly provided by Dr. Jerry Shay (UT Southwestern, Dallas TX). HCEC cells were maintained in DMEM media with 2% calf serum, 25 ng/ml EGF (Sigma Aldrich, St. Louis, MO), 2 μg/ml transferrin (Sigma Aldrich, St. Louis, MO), 10 μg/ml insulin (Sigma Aldrich), 5 nM sodium selenite (Sigma Aldrich, St. Louis, MO), 1 μg/ml hydrocortisone (Sigma Aldrich), and 50 μg/ml gentamicin (Gemini Bio-products, West Sacramento, CA). HCEC cells were grown at 37 °C on Primaria flasks (Corning Inc., Conring NY) placed in chambers purged with 93% nitrogen 5% carbon dioxide and 2% oxygen gas. All other cell lines were maintained in DMEM with 4 mM L-glutamine, 10% FBS, and 1x penicillin/streptomycin at 37 °C and 5% carbon dioxide.

### Vectors

RNAi experiments were performed using pLKO.1 vectors obtained through the University of Minnesota Genomics Center (UMGC) partnership with the Open Access Program from Open Biosystems. The pLKO.1 vector with the oligo ID (TRCN0000059768) was used to generate DLD1 shRNA3 cells and the vector with oligo ID (TRCN0000059772) was used to generate DLD1 shRNA7 cells. Non-silencing control vectors were obtained in the same manner. The lentiviral packaging vector psPAX2 was a gift from Didier Trono (Addgene plasmid #12260) and viral envelope encoding vector pCMV-VSV-G was a gift from Bob Weinberg (Addgene plasmid #8454). The lentiCRISPR v2 vector used to generate TM9SF2 knockout cells was a gift from Feng Zhang (Addgene plasmid # 52961).

### ShRNA knockdown cells

DLD1 stable TM9SF2 knockdown cells were created by transduction with lentiviral particles encoding TM9SF2 shRNAs followed by puromycin selection. Virus production was performed using standard 293 T packaging protocols and the pLKO.1 vectors.

### CRISPR/Cas9 knockout cells

CRISPR lentiviral constructs targeting TM9SF2 were generated by annealing together primers for sgTM9SF2 CRISPR1, sgTM9SF2 CRISPR2, and sgTM9SF2 CRISPR6 and cloning the annealed product into lentiCRISPR v2 as described previously^49^. The sgRNA primer sequences are listed in Supplementary Table S1. The sgRNAs were designed to specifically target coding regions of the *TM9SF2* gene using the CRISPR design tool from Dr. Feng Zhang’s laboratory (http://crisper.mit.edu/). After transducing cells with *TM9SF2* targeting CRISPR/Cas9 lentiviral particles, single cell puromycin-resistant clones were isolated by limiting dilution. DNA was extracted from single cell clones for PCR amplification of the genomic loci targeted by CRISPR/Cas9. After PCR, the amplicons were purified and subsequently sequenced. Sequencing data from these clones was then used for Tracking of Indels by Decomposition (TIDE) analysis and mutants were confirmed by immunoblot with an anti-TM9SF2 antibody (Sigma Aldrich, St. Louis, MO, Catalog # HPA005657) using a standard Western Blotting protocol.

### Soft Agar assay and cell proliferation by trypan blue exclusion

For soft agar assays, cells were grown in between layers of 0.5% Sea Plaque Low Melt Agarose (Lonza Cat. # 50101) in DMEM supplemented with 10% FBS and 1x penicillin/streptomycin for 10 days. Colonies were fixed, stained with crystal violet, and imaged. Colony counts were analyzed using ImageJ software. Equal numbers of HT-29 control and TM9SF2 knockout cells were plated in triplicate wells of 24-well culture plates and allowed to adhere for 24 hours. Cells were detached using trypsin and, after adding trypan blue, counted with an automated cell counter (Thermo Fisher Scientific, Waltham MA). The total number of live cells was determined for each well and the average number of viable cells for each triplicate was plotted for days 1 through 4.

### RNA extraction and qRT-PCR

Total RNA was extracted using the RNeasy Plus mini kit (Qiagen, Germantown, MD) according to the manufacturer’s protocol. Total RNA concentration and quality were measured using the BioTek Epoch microplate spectrophotometer (BioTek, Winooski, VT). One microgram of total RNA was reverse transcribed using the High Capacity cDNA reverse transcription kit (Applied Biosystems, Foster City, CA). RT-qPCR was then performed in triplicate using diluted cDNA, FastStart Essential DNA Green Master mix (Roche, Basel, Switzerland), and either *ELF1* or *TM9SF2* specific primers. Samples were run in the LightCycler 96 (Roche, Basel, Switzerland). Data were normalized to human beta actin and fold change was calculated using the delta-delta Ct method. Primer sequences are listed in Supplementary Table S1.

### Xenograft model

Athymic nude mice were obtained from The Jackson Laboratory (Stock No. 002019) and protocols were approved by the University of Minnesota’s Institutional Animal Care and Use committee. Five million HT-29 control and TM9SF2 knockout cells were resuspended in 200 µL of DMEM media and subcutaneously injected into the rear flank. Each mouse carried duplicate tumors on the left and right flank. Tumor volume was measured with digital calipers every other day and tumor volume was calculated using the formula: volume = (width)^2^ × (length)/2, where the width is the smaller of the measurements. All animal experimental protocols were approved by the University of Minnesota’s Institutional Animal Care and Use Committee (IACUC Protocol ID: 1702-34600A). All experiments were performed in accordance with the American Association for Accreditation of Laboratory Animal Care (AAALAC) relevant guidelines and regulations for performing live vertebrate experiments.

### Gene expression analysis

Oncoprint data showing the genomic alterations in TM9SF2 were generated using cBioPortal (version 1.12.1; http://www.cbioportal.org/index.do) using both RNA-seq and Microarray data from Colorectal Adenomacarcinoma (TCGA, Provisional)^27,28^. cBioPortal enrichment analysis revealed that *TM9SF2* and *ELF1* were highly positively correlated as measured by Pearson’s or Spearman’s correlation.

### RNA sequencing

Matched frozen tumor and normal samples were acquired from 44 patients (88 samples total) from the University of Minnesota’s Tissue Repository. All samples were de-identified. For RNA-seq of patient samples, total RNA was extracted using a previously established protocol^50,51^. Purified RNA was submitted to the University of Minnesota Genomics Center for library preparation and sequencing. A quality check of raw sequence data (FASTQ files) was performed using FastQC software (https://www.bioinformatics.babraham.ac.uk/projects/fastqc/) to assess overall sequence quality, GC content, adaptor content, etc. Quality trimming was performed to remove sequence adaptors and low-quality bases using Trimmomatic with a 3 bp sliding window trimming from 3′ end requiring minimum Q16 (phred33)^52^. FastQC was run on the resulting trimmed FASTQ files to ensure good quality of sequences. The paired-end reads were mapped to NCBI v38 *H. sapiens* reference genome using HISAT2, resulting in average alignment rate of 87.11% overall for 88 samples^53^. SAMtools was used for sorting and indexing the aligned bam files. After alignment, featureCounts was used to generate gene abundance, and Cuffnorm was used to generate an FPKM expression table^54^. We used ensembl web-service from biomaRt package in R to map gene transcripts (ensemble gene id) to HGNC gene symbols^55^.

RNA-seq of HT-29 knockout cells: 50 bp FastQ paired-end reads (n = 8.8 million per sample) were trimmed using Trimmomatic (v 0.33) enabled with the optional “-q” option; 3 bp sliding-window trimming from 3′ end requiring minimum Q30. Quality control checks on raw sequence data for each sample were performed with FastQC. Read mapping was performed via HISAT2 (v2.0.2) using the UCSC human genome (hg38) as reference. Gene quantification was done via Cuffquant for FPKM values and Feature Counts for raw read counts. Differentially expressed genes were identified using the edgeR (negative binomial) feature in CLCGWB (Qiagen, Valencia, CA) using raw read counts. We filtered the generated list based on a minimum 2X Absolute Fold Change and Bonferroni corrected p < 0.05. These filtered genes were then imported into Ingenuity Pathway Analysis Software (Qiagen, Valencia, CA) for pathway identification.

### Chromatin Immunoprecipitation and qPCR

DLD1 cells were plated at 3 × 10^6^ cells per 10 cm plate in standard fully supplemented DMEM overnight. ChIP was performed using an ELF-1 specific antibody (Santa Cruz, SC-631) or a non-specific IgG rabbit isotype control using Protein G magnetic beads (Active Motif). Analysis was performed using previously described methods^56^. qPCR data are presented as Fold enrichment relative to the negative (IgG) sample. Primers are listed in Supplemental Table S1.

### Patient Prognoses and Survival

The Staub cohort of 62 CRC patient samples used for transcriptome analysis by microarray (GSE12945) was previously described^57^. Patients were grouped by disease stage and the mean expression value of *TM9SF2* in each stage was plotted using box plots. Statistical significance between groups were determined using one-way ANOVA with a Tukey’s *post hoc* test. Kaplan Meier curves depicting relapse-free survival (RFS) probability were generated using the R2 genomics analysis and visualization platform (http://hgserver1.amc.nl/). The Marisa *et al*. cohort consisting of 566 samples was used for RFS analysis with two groups (high vs. low) *TM9SF2* mRNA expression divided based on optimal expression separation. RFS groups were compared using the log-rank test. The same method was used when analyzing the 355-patient cohort provided by Sieber and Smith (GSE1433 and GSE175)^41,42^.

## Electronic supplementary material

Supplemental Information

Supplementary Data

## References

[1] Vogelstein B (1988). Genetic alterations during colorectal-tumor development. The New England journal of medicine.

[2] Tanaka T (2009). Colorectal carcinogenesis: Review of human and experimental animal studies. J Carcinog.

[3] Cancer Genome Atlas, N (2012). Comprehensive molecular characterization of human colon and rectal cancer. Nature.

[4] Haan JC (2014). Genomic landscape of metastatic colorectal cancer. Nat Commun.

[5] Starr TK (2009). A transposon-based genetic screen in mice identifies genes altered in colorectal cancer. Science.

[6] Starr TK (2011). A Sleeping Beauty transposon-mediated screen identifies murine susceptibility genes for adenomatous polyposis coli (Apc)-dependent intestinal tumorigenesis. Proceedings of the National Academy of Sciences of the United States of America.

[7] Takeda H (2015). Transposon mutagenesis identifies genes and evolutionary forces driving gastrointestinal tract tumor progression. Nature genetics.

[8] Abbott KL (2015). The Candidate Cancer Gene Database: a database of cancer driver genes from forward genetic screens in mice. Nucleic Acids Res.

[9] Than BL (2014). The role of KCNQ1 in mouse and human gastrointestinal cancers. Oncogene.

[10] Than BLN (2017). CFTR is a tumor suppressor gene in murine and human intestinal cancer. Oncogene.

[11] Seshagiri S (2012). Recurrent R-spondin fusions in colon cancer. Nature.

[12] Pruvot B (2010). Comparative analysis of nonaspanin protein sequences and expression studies in zebrafish. Immunogenetics.

[13] Chluba-de Tapia J, de Tapia M, Jaggin V, Eberle AN (1997). Cloning of a human multispanning membrane protein cDNA: evidence for a new protein family. Gene.

[14] He P (2009). High-throughput functional screening for autophagy-related genes and identification of TM9SF1 as an autophagosome-inducing gene. Autophagy.

[15] Zaravinos A, Lambrou GI, Boulalas I, Delakas D, Spandidos DA (2011). Identification of common differentially expressed genes in urinary bladder cancer. PloS one.

[16] Chang H (2011). Identification of genes associated with chemosensitivity to SAHA/taxane combination treatment in taxane-resistant breast cancer cells. Breast cancer research and treatment.

[17] Oo HZ (2014). Identification of novel transmembrane proteins in scirrhous-type gastric cancer by the Escherichia coli ampicillin secretion trap (CAST) method: TM9SF3 participates in tumor invasion and serves as a prognostic factor. Pathobiology.

[18] Lozupone F (2009). The human homologue of Dictyostelium discoideum phg1A is expressed by human metastatic melanoma cells. EMBO reports.

[19] Lozupone F (2015). TM9SF4 is a novel V-ATPase-interacting protein that modulates tumor pH alterations associated with drug resistance and invasiveness of colon cancer cells. Oncogene.

[20] Starr TK, Largaespada DA (2005). Cancer gene discovery using the Sleeping Beauty transposon. Cell Cycle.

[21] Dupuy AJ, Akagi K, Largaespada DA, Copeland NG, Jenkins NA (2005). Mammalian mutagenesis using a highly mobile somatic Sleeping Beauty transposon system. Nature.

[22] Collier LS, Carlson CM, Ravimohan S, Dupuy AJ, Largaespada DA (2005). Cancer gene discovery in solid tumours using transposon-based somatic mutagenesis in the mouse. Nature.

[23] Copeland NG, Jenkins NA (2010). Harnessing transposons for cancer gene discovery. Nature reviews. Cancer.

[24] Newberg JY, Mann KM, Mann MB, Jenkins NA, Copeland NG (2018). SBCDDB: Sleeping Beauty Cancer Driver Database for gene discovery in mouse models of human cancers. Nucleic Acids Res.

[25] March HN (2011). Insertional mutagenesis identifies multiple networks of cooperating genes driving intestinal tumorigenesis. Nature genetics.

[26] Forbes SA (2017). COSMIC: somatic cancer genetics at high-resolution. Nucleic Acids Res.

[27] Cerami E (2012). The cBio cancer genomics portal: an open platform for exploring multidimensional cancer genomics data. Cancer discovery.

[28] Gao J (2013). Integrative analysis of complex cancer genomics and clinical profiles using the cBioPortal. Science signaling.

[29] Roig AI (2010). Immortalized epithelial cells derived from human colon biopsies express stem cell markers and differentiate *in vitro*. Gastroenterology.

[30] Subramanian A (2005). Gene set enrichment analysis: a knowledge-based approach for interpreting genome-wide expression profiles. Proceedings of the National Academy of Sciences of the United States of America.

[31] Stanton RC (2012). Glucose-6-phosphate dehydrogenase, NADPH, and cell survival. IUBMB Life.

[32] Ho HY (2000). Enhanced oxidative stress and accelerated cellular senescence in glucose-6-phosphate dehydrogenase (G6PD)-deficient human fibroblasts. Free Radic Biol Med.

[33] Longo L (2002). Maternally transmitted severe glucose 6-phosphate dehydrogenase deficiency is an embryonic lethal. EMBO J.

[34] Widau RC, Jin Y, Dixon SA, Wadzinski BE, Gallagher PJ (2010). Protein phosphatase 2A (PP2A) holoenzymes regulate death-associated protein kinase (DAPK) in ceramide-induced anoikis. The Journal of biological chemistry.

[35] Powell JA (2017). Targeting sphingosine kinase 1 induces MCL1-dependent cell death in acute myeloid leukemia. Blood.

[36] Adada MM (2015). Intracellular sphingosine kinase 2-derived sphingosine-1-phosphate mediates epidermal growth factor-induced ezrin-radixin-moesin phosphorylation and cancer cell invasion. FASEB J.

[37] Shida D, Takabe K, Kapitonov D, Milstien S, Spiegel S (2008). Targeting SphK1 as a new strategy against cancer. Curr Drug Targets.

[38] Kim WJ (2008). Mutations in the neutral sphingomyelinase gene SMPD3 implicate the ceramide pathway in human leukemias. Blood.

[39] Consortium EP (2012). An integrated encyclopedia of DNA elements in the human genome. Nature.

[40] Marisa L (2013). Gene expression classification of colon cancer into molecular subtypes: characterization, validation, and prognostic value. PLoS Med.

[41] Jorissen RN (2009). Metastasis-Associated Gene Expression Changes Predict Poor Outcomes in Patients with Dukes Stage B and C Colorectal Cancer. Clinical cancer research: an official journal of the American Association for Cancer Research.

[42] Smith JJ (2010). Experimentally derived metastasis gene expression profile predicts recurrence and death in patients with colon cancer. Gastroenterology.

[43] Froquet R (2012). TM9/Phg1 and SadA proteins control surface expression and stability of SibA adhesion molecules in Dictyostelium. Molecular biology of the cell.

[44] Perrin J (2015). TM9 family proteins control surface targeting of glycine-rich transmembrane domains. Journal of cell science.

[45] Ordonez C, Screaton RA, Ilantzis C, Stanners CP (2000). Human carcinoembryonic antigen functions as a general inhibitor of anoikis. Cancer research.

[46] Ilantzis C, DeMarte L, Screaton RA, Stanners CP (2002). Deregulated expression of the human tumor marker CEA and CEA family member CEACAM6 disrupts tissue architecture and blocks colonocyte differentiation. Neoplasia.

[47] Lai M (2017). Complete Acid Ceramidase ablation prevents cancer-initiating cell formation in melanoma cells. Scientific reports.

[48] Goni FM, Alonso A (2002). Sphingomyelinases: enzymology and membrane activity. FEBS letters.

[49] Sanjana NE, Shalem O, Zhang F (2014). Improved vectors and genome-wide libraries for CRISPR screening. Nat Methods.

[50] Burns MB (2013). APOBEC3B is an enzymatic source of mutation in breast cancer. Nature.

[51] Burns MB, Lynch J, Starr TK, Knights D, Blekhman R (2015). Virulence genes are a signature of the microbiome in the colorectal tumor microenvironment. Genome Med.

[52] Bolger AM, Lohse M, Usadel B (2014). Trimmomatic: a flexible trimmer for Illumina sequence data. Bioinformatics.

[53] Kim D, Langmead B, Salzberg SL (2015). HISAT: a fast spliced aligner with low memory requirements. Nat Methods.

[54] Liao Y, Smyth GK, Shi W (2014). featureCounts: an efficient general purpose program for assigning sequence reads to genomic features. Bioinformatics.

[55] Durinck S, Spellman PT, Birney E, Huber W (2009). Mapping identifiers for the integration of genomic datasets with the R/Bioconductor package biomaRt. Nat Protoc.

[56] Chan SC (2015). Targeting chromatin binding regulation of constitutively active AR variants to overcome prostate cancer resistance to endocrine-based therapies. Nucleic Acids Res.

[57] Staub E (2009). An expression module of WIPF1-coexpressed genes identifies patients with favorable prognosis in three tumor types. J Mol Med (Berl).

