# Supplementary material for: Transposon mutagenesis screen in mice identifies TM9SF2 as a novel colorectal cancer oncogene
Source: Sci Rep. 2018 Oct 17;8:15327. doi: 10.1038/s41598-018-33527-3 (PMC6193042; doi:10.1038/s41598-018-33527-3)
Supplement: Supplementary file 1 — Supplemental Information [file 41598_2018_33527_MOESM1_ESM.pdf]

Transposon mutagenesis screen in mice identifies *TM9SF2* as a novel colorectal cancer oncogene

Running Title: *TM9SF2* is a novel colorectal cancer driver gene.

Authors: Christopher R. Clark [a], Makayla Maile [a], Patrick Blaney [a], Stefano R. Hellweg [a], Anna Strauss [a], Wilaiwan Durose [a], Sambhawa Priya [d,e], Juri Habicht [a], Michael B. Burns [f], Ran Blekhman [d,e], Juan E. Abrahante [c], Timothy K. Starr [a,b]

Supplement 1: TM9SF2 knockdown reduces anchorage independent growth.

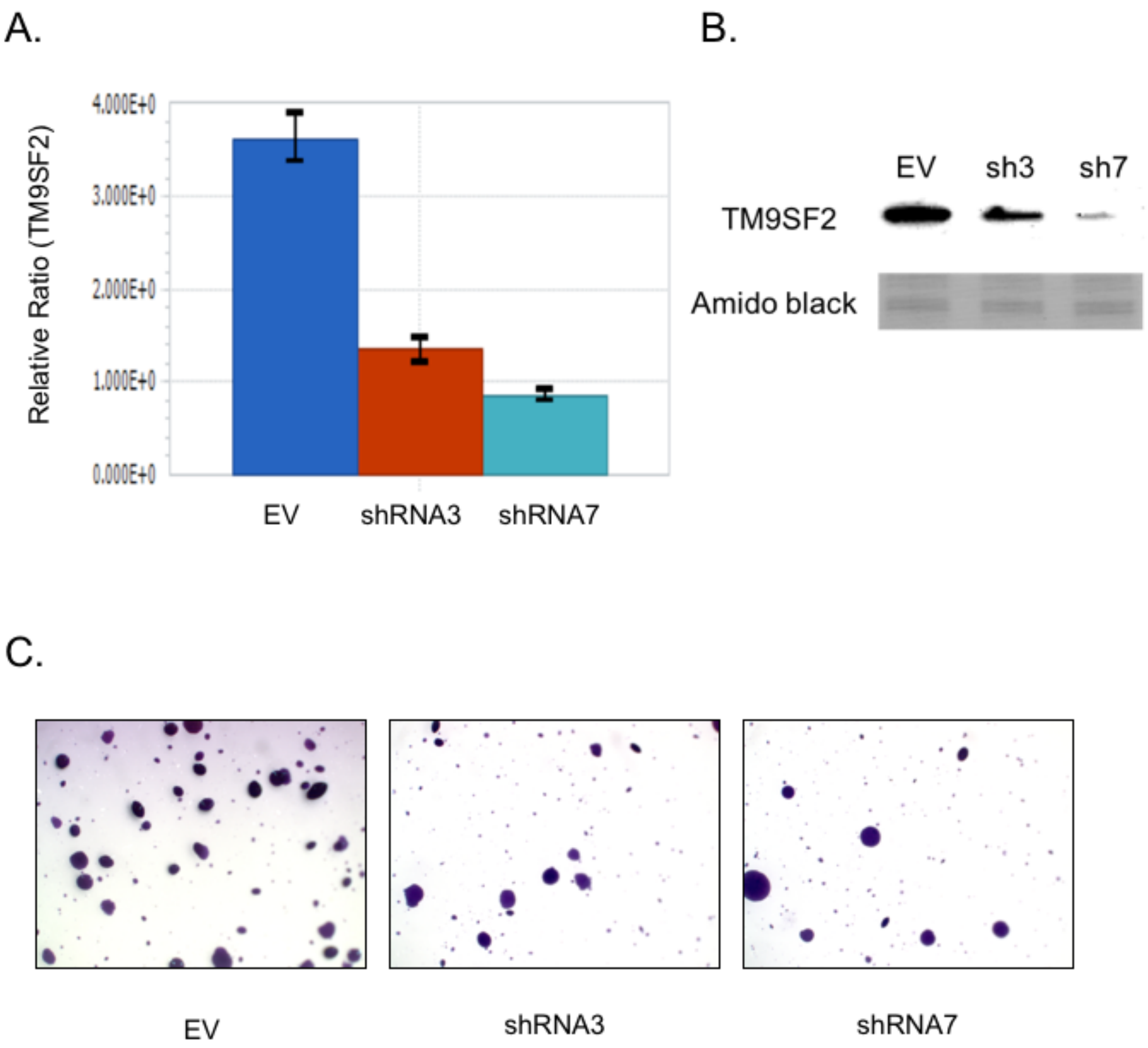

Supplemental 1: *TM9SF2* knockdown reduces anchorage-independent growth in DLD1 cells. A, quantification of *TM9SF2* mRNA levels in DLD1 cells transduced with lentiviral particles carrying *TM9SF2* shRNA. B, Western blot confirmation of TM9SF2 knockdown in DLD1 cells. C, Images of colonies stained with crystal violet ten days post plating.

Supplement 2: TM9SF2 knockout schema and knockout confirmation

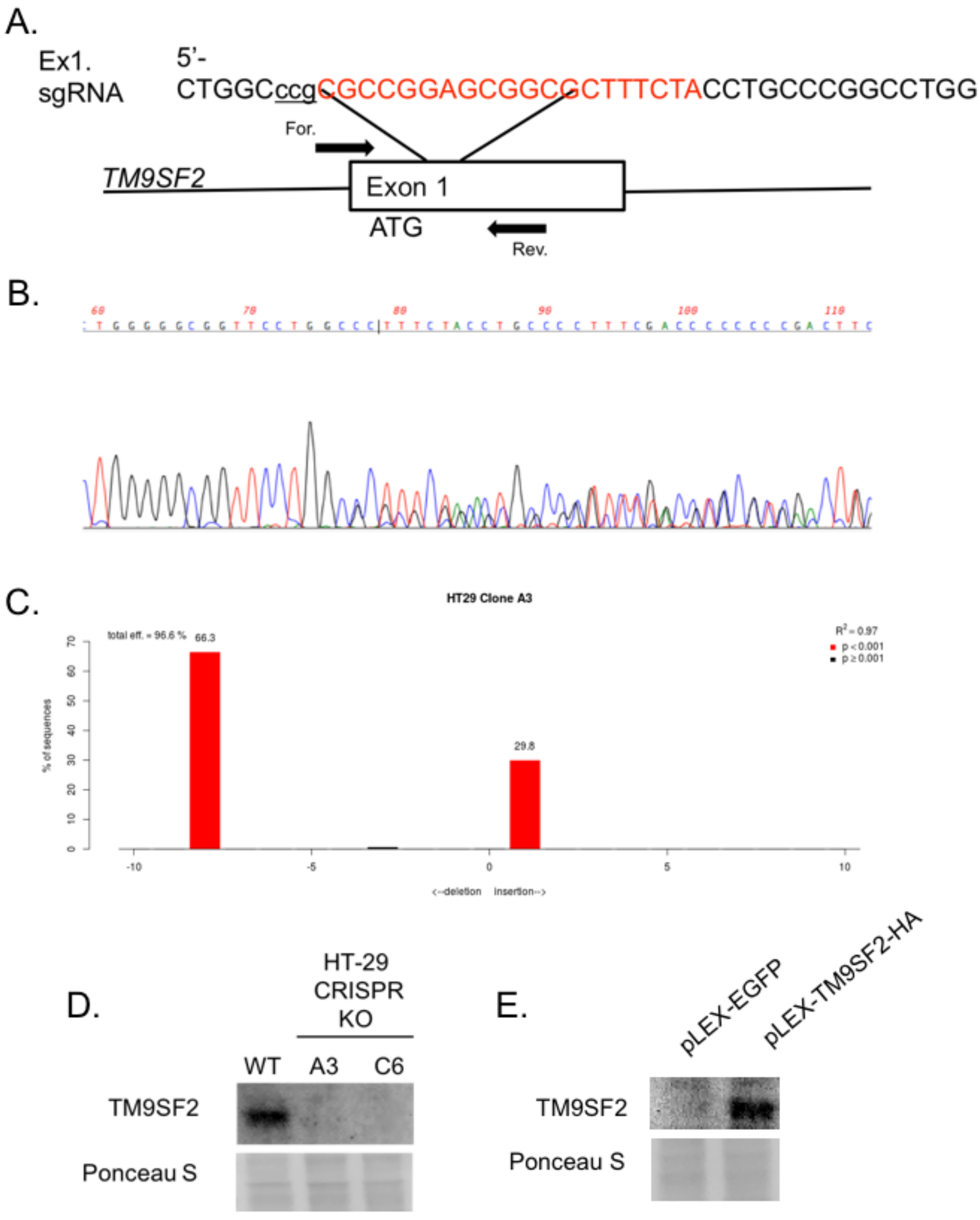

Supplemental 2: A, schematic of *TM9SF2* gene editing with CRISPR/Cas9 lentiV2. Underlined nucleotides represent the protospacer adjacent motif (PAM) sequence following the DNA sequence target by the Cas9 nuclease (DNA sequence in red font). B, Representative sequence trace decomposition by TIDE analysis. Shown is the predicted mutant sequence after CRISPR/Cas9 editing and the indel spectrum determined by tide (Fig C). D, western blot analysis for TM9SF2 protein expression in single cell HT-29 knockout clones. E, western blot analysis for TM9SF2 protein expression in HCT116 overexpression cells.

Supplement 3: ENCODE H3K4me3 and ELF1 Chip-Seq peaks in HCT116 cells.

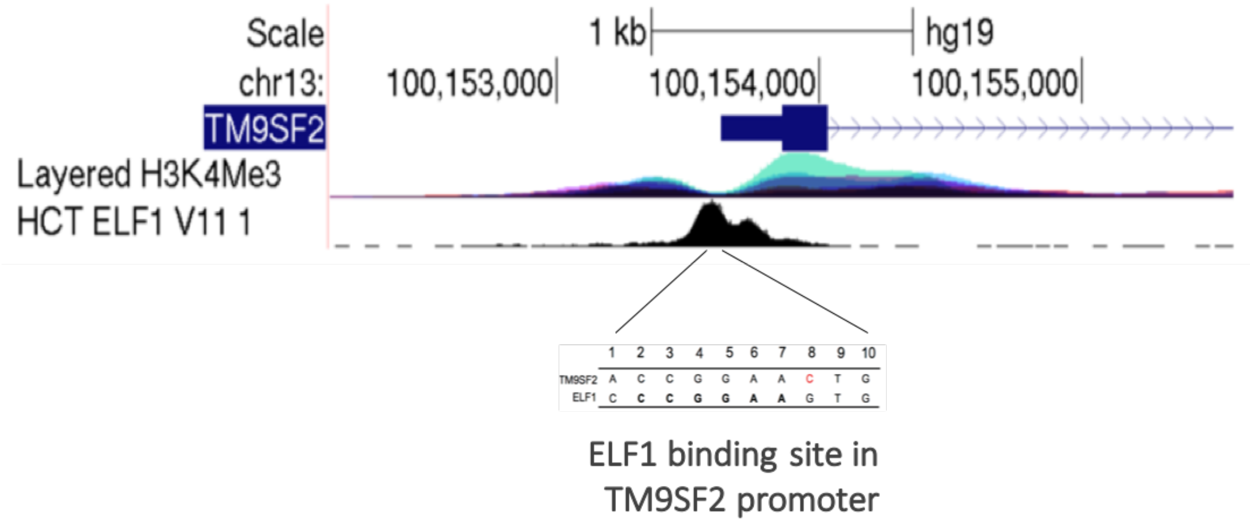

Supplemental 3: Gene track view of Chip-Seq data at the TM9SF2 locus (Chr 13). The read density after H3K4me4 (middle track) and ELF-1 immunoprecipitations are show (bottom track). Also pictured is the ELF1 binding motif in the TM9SF2 promoter region. Data is from the UCSC genome browser (GRch37/hg19).
